# Advancing the Science on Unexpected Episodes of Clarity and Lucidity in People With Dementia

**DOI:** 10.1093/geroni/igab046.176

**Published:** 2021-12-17

**Authors:** Joan Griffin, Basil Eldadah

## Abstract

People with late-stage Alzheimer’s disease and related dementias (ADRD) have been reported, largely by way of anecdote, to exhibit unexpected episodes of spontaneous, meaningful, and relevant communication or behavior. These episodes of lucidity (EL) are characterized by spontaneous mental clarity in people living with dementia (PLWD) who are assumed to have lost coherent cognitive capacity. Given the transient nature and limited understanding of underlying mechanisms responsible for this phenomenon, these episodes are frequently overlooked and have received little scientific attention. Few studies have documented EL among PLWD with precision; scientific understanding is limited to anecdotes and case studies, which have not operationalized EL. Thus, there is a critical need for an evidence-based understanding and systematic operationalization of EL. Precise and robust operationalizations of EL will allow future research to assess if EL has different effects on ADRD prognosis or alters how family members manage and adapt to ADRD progression in their care recipient. The National Institute on Aging (NIA) has funded six studies to advance the scientific understanding of EL in dementia. These studies use a variety of methodological approaches to capture EL experiences, and together, they will provide evidence-based operational definitions of EL, novel approaches for measurement of this phenomenon, and estimates of its prevalence. This symposium will provide an overview of the funded studies and three different methodological approaches that are being used to better operationalize and understand EL.

